# Molecular mechanisms underlying the impact of muscle fiber types on meat quality in livestock and poultry

**DOI:** 10.3389/fvets.2023.1284551

**Published:** 2023-11-22

**Authors:** Meijie Mo, Zihao Zhang, Xiaotong Wang, Wenjin Shen, Li Zhang, Shudai Lin

**Affiliations:** College of Coastal Agricultural Sciences, Guangdong Ocean University, Zhanjiang, Guangdong, China

**Keywords:** livestock and poultry, skeletal muscle, meat quality, muscle fiber types, mechanism

## Abstract

In the past, the primary emphasis of livestock and poultry breeding was mainly on improving the growth rate, meat production efficiency and disease resistance. However, the improvement of meat quality has become a major industrial focus due to the ongoing advancements in livestock and poultry breeding. Skeletal muscles consist of multinucleated myofibers formed through the processes of myoblast proliferation, differentiation and fusion. Muscle fibers can be broadly classified into two main types: slow-twitch (Type I) and fast-twitch (Type II). Fast-twitch fibers can be further categorized into Type IIa, Type IIx, and Type IIb. The proportion of Type I and Type IIa muscle fibers is positively associated with meat quality, while the presence of Type IIb muscle fibers in skeletal muscle tissue is inversely related to meat quality. Consequently, muscle fiber composition directly influences meat quality. The distribution of these fiber types within skeletal muscle is governed by a complex network, which encompasses numerous pivotal regulators and intricate signaling pathways. This article aims to succinctly outline the parameters utilized for assessing meat quality, elucidate the relationship between muscle fiber composition and meat quality as well as elaborate on the relevant genetic factors and their molecular mechanisms that regulate muscle fiber types in livestock and poultry. This summary will enrich our comprehension of how to improve meat quality in livestock and poultry, providing valuable insights for future improvements.

## 1 Introduction

Meat quality encompasses various characteristics related to the appearance and palatability of both fresh meat and processed meat, including color, texture structure, water-holding capacity, tenderness, and flavor of the meat (1). These attributes play a pivotal role in shaping the overall meat-eating experience, are thus essential indicators for meat quality assessment. Traditional methods for evaluating meat quality have predominantly relied on physical and chemical techniques. However, these methods come with several drawbacks, such as time-consuming, labor requirements, potential meat damage, high costs, and extended sample preparation times. Additionally, they may be suitable only for laboratory assessments and not feasible for using in commercial poultry processing facilities (2, 3). Therefore, there has been a shift toward developing automated commercial testing systems to monitor meat quality. These systems not only measure meat quality but also help reduce economic losses, thereby enhancing the overall economic efficiency of the poultry industry (2).

Skeletal muscle, the predominant muscle type in animals, is primarily composed of two types of muscle fibers: red (slow-twitch) fibers and white (fast-twitch) fibers. The fiber type composition within skeletal muscle directly influences meat quality attributes, such as color, tenderness, post-slaughter pH, and various other key factors (4). Extensive research has revealed a noteworthy positive correlation between heightened myoglobin content in red fibers and enhanced meat tenderness and palatability. Conversely, white fibers, with their lower myoglobin content, result in a relatively coarser meat texture (5). Conventional production practices have typically employed strategies such as feeding management, feed formulation, and exercise to modulate muscle fiber composition and consequently influence meat quality (6, 7). In recent years, advances in molecular biology, genetics, and nutrition have deepened our understanding of the intricate relationship between muscle fiber type and meat quality. By employing techniques such as genomics, metabonomics and other methods to elucidate the fine molecular mechanisms that affect skeletal muscle development, researchers strive to change the relative proportion of muscle fiber types in order to improve meat quality (8, 9).

During the development and differentiation of skeletal muscle, a series of processes are dependent on the regulation of genes related to myogenesis and related signaling pathways. Research has demonstrated the involvement of Myogenic Regulatory Factors (*MRFs*), Myostatin (*Mstn*), the paired box (*Pax*) family, and Forkhead Box Transcription Factors (FoxO) signaling pathways in regulating the fundamental processes of skeletal muscle development (10). Numerous genetic factors regulated the entire skeletal muscle development process which primarily involves somite proliferation and differentiation, myoblast proliferation and differentiation, myotube fusion, and the formation of muscle fibers (11). Moreover, non-coding RNAs, including microRNAs (miRNAs), long non-coding RNAs (lncRNAs), and circular RNAs (circRNAs), have also been found to participate in these processes (10). Currently, significant progress has already been achieved in the study of genes and signaling pathways that influence muscle fiber types in livestock and poultry. Through employing transcriptomics and gene editing techniques, numerous genes influencing muscle fiber types have been successfully identified and validated. Furthermore, a previous study has illuminated the roles of multiple signaling pathways in regulating muscle fiber types (12). These findings offer novel insights and methods for improving livestock production and meat quality. Nonetheless, it's important to note that regulatory mechanisms controlling muscle fiber types can vary among different animal breeds and species, underscoring the need for further comparative research.

## 2 Meat quality and its evaluation indicators

### 2.1 Meat quality

Meat quality encompasses various attributes associated with meat. From the perspective of food science, meat quality refers to specific characteristics related to the appearance and palatability of both fresh and processed meat. These characteristics includes color, texture structure, water-holding capacity, tenderness, and the overall flavor of the meat (1).

### 2.2 Indicators and methods for evaluating meat quality

The assessment criteria for meat quality typically encompass parameters such as color, pH, water-holding capacity, tenderness, flavor, and so on. The content and composition of different muscle fiber types, which form the fundamental structural unit of muscles, directly influence the aforementioned meat quality indicators, especially the diameter, type, and quantity of muscle fibers (13).

#### 2.2.1 Meat color

The color of meat is primarily determined by the concentrations of myoglobin and hemoglobin present within muscle fibers. Muscle fibers of Type I and Type IIa possess higher myoglobin levels, resulting in a bright red appearance, while Type IIb muscle fibers exhibit lower myoglobin levels, leading to a paler meat color (14). Generally, research indicates a positive correlation between the intensity of redness of meat and the proportion of Type I and Type IIa muscle fibers within the muscle structure (15). Meat color can be measured using a variety of techniques, including sensory evaluation, colorimetric measurement, myoglobin measurement, computer vision measurement, and multispectral vision systems. The more conventional methods among them include sensory evaluation and colorimetric measurement. In the past, measuring meat color typically relied on a meat color score, which was simple, convenient, but very subjective. The meat's freshness and quality can be directly detected by the myoglobin determination method, which is easy, quick, and accurate, but cannot be used with other meat quality indicators. Despite being highly accurate and repeatable, colorimeters cannot directly reflect the freshness and quality of meat. Computer vision has the advantages of high accuracy, fast speed, strong objectivity, and non-contact (16), but it also has expensive equipment costs, complicated data processing, and limited applicability (17). A multispectral vision system can automate, do non-destructive testing, collect more complete and accurate information, and increase efficiency by concurrently measuring the reflection or absorption of multiple wavelengths of light. However, it is relatively expensive to operate and maintain the instruments and equipment, requiring the use of skilled technical employee (18). The common techniques for assessing meat color are shown in Table 1.

**Table 1:** Common methods for evaluating meat color.

**Common methods**	**Principle**	**Advantages**	**Disadvantages**	**References**
Myoglobin assay	Determining meat color based on the state of Feions in myoglobin.	Accurate results	Tedious sample preparation process	(19)
Colorimeter	Assessing color types, brightness, hue, intensity, and saturation through color, brightness, and hue measurements.	High precision, objective	Results vulnerable to meat sample thickness	(20)
Computer vision	Utilizing color theory and statistical analysis.	Accurate results	Requires consistent lighting conditions	(20)
Multispectral vision system	Mapping multispectral pixel-wise information to CIELab values with a PIM.	Non-pollution	Cannot directly transformed to the sRGB	(18)

CIELab, color space is an international standard for color; PIM, photometric imaging model; sRGB, standardized color space.

#### 2.2.2 pH value

Meat's pH value can be accurately measured through the utilization of a pH meter or visible and near-infrared reflectance spectroscopy (Vis/NIRS) (21). The pH value of muscle tissue in livestock and poultry is influenced by the glycogen content and the rate of adenosine triphosphate (ATP) degradation rate within the animal's muscles at the time of slaughter. Type IIb muscle fibers, known for their glycolytic properties, contain high level of glycogen and display significant ATP enzyme activity, resulting in rapid glycolysis and a swift decrease in pH value. The pH value could be served as an indicator of the glycogenolytic capacity in muscle after slaughter (22). Excessively low pH values may cause the denaturation of muscle fiber proteins, leading to the occurrence of PSE (Pale, Soft, Exudative) meat, characterized by its pale color, water loss, and soft texture (23). For example, 1 h after the death of Duroc × Landrace × Taking Yorkshire crossed pigs, the pH value of PSE meat is < 5.8, and the pH value of RFN (Red, Firm, and Non-Exudative) is ≥ 5.8 (23, 24). Moreover, attributes related to muscle quality, such as water-holding capacity, tenderness and flavor, could suffer negative effects (25). Therefore, a reduction in the proportion of Type IIb muscle fibers can contribute to stabilizing the pH value of the meat and reducing the likelihood of PSE meat occurrence (21).

#### 2.2.3 Water-holding capacity

Water-holding capacity refers to the muscle proteins' ability to retain internal moisture and is often assessed by drip loss measurements. Rapid glycolysis occurring in glycolytic muscle fibers results in a quick pH decline, triggering myofibril contraction within the muscle fibers, consequently expelling water from the muscle and reducing water-holding capacity. The content of Type I muscle fibers has been shown to negatively correlate with drip loss (26), while Type IIb muscle fibers has been observed to positively associate with drip loss (27). Water-holding capacity can be carried out using techniques such as pressure weighing, drip loss assessment or the cooking yield method (Table 2). The pressure weighing method can accurately measure meat's water content, but the process of operation is relatively complicated and requires specific equipment and technical support. By monitoring meat samples' drip loss over a predetermined amount of time, the drip loss method assesses the water retention rate of the meat samples. The operation is straightforward, but the accuracy of the measurement data might be impacted by ambient variables such as temperature and humidity. The cooking yield method estimates the water content of meat by measuring its water loss during the cooking process. It has the benefit of being able to intuitively reflect the flavor and texture of meat, making it suitable for both restaurant and home cooking. However, it will change certain characteristics of meat and has limited applicability (28). Hyperspectral imaging technology is a technique for obtaining surface details of an object by collecting the spectral reflection or emission information of the object in multiple narrow bands. It has the characteristics of high resolution, high sensitivity, non-contact, and multi-dimensional information. However, data processing and analysis are difficult and expensive, because they are influenced by factors such as lighting conditions and atmospheric variables (29).

**Table 2:** Common methods for evaluating meat water-holding capacity.

**Common methods**	**Principle**	**Advantages**	**Disadvantages**	**References**
Pressure weighing method	Applying an external force to alter the water-holding structure of the meat sample and subsequently measuring water loss.	Simple and easy to perform	Limited sample size, less representative	(28)
Drip loss method	Measuring the liquid loss within the protein system of the meat sample under the influence of gravity alone.	Less influenced by external factors	Structural damage to the meat, reducing accuracy	(28)
Cooking yield method	Measuring the degree of water loss through heating.	Predicting juiciness of the meat sample	-	(28)
HIS	Obtaining spatial and spectral information from object pixels to facilitate both qualitative and quantitative analysis.	Rapid, accurate, and Non-destructive detection	High instrument cost	(29)

“-” Indicates no record; HIS, hyperspectral imaging technique.

#### 2.2.4 Tenderness

As an essential sensory characteristic of meat quality, tenderness is often assessed through measuring the shear force. Actually, meat tenderness can be evaluated using probe testing, shear force testing, or texture analyzer methods (Table 3). The strength and hardness of muscle fibers can be directly and objectively reflected by shear force testing, a typical technique for determining meat tenderness, but it is affected by operational technology and equipment accuracy. When cutting a meat sample through a texture analyzer, the probe test records the force exerted by the cutting tool, and it uses the measured peak shear force (the maximum value of force) as the meat sample's tenderness value. Using this method, a large number of samples can be measured fast, but at a considerable expense. The main principle of texture analyzer testing is to obtain the texture information of an object by analyzing the morphological features of its surface microstructure. It can provide detailed information about texture, which helps to deeply understand the structural characteristics of meat quality. However, the equipment is complex and pricey, requiring specialized knowledge and skills for operation and analysis (30).

**Table 3:** Common methods for evaluating meat tenderness.

**Common methods**	**Principle**	**Advantages**	**Disadvantages**	**References**
Shear force testing	Measuring the force required to slice through a standardized blade with a meat sample.	Objective measurement	Tedious sample preparation process, difficulty in controlling core temperature of the sample	(30)
Probe testing	Quantifying the torque/angle trajectory to describe texture features.	Rapid testing process	High equipment cost	(30)
Texture analyzer testing	Penetrating the meat sample to determine its extreme points and subsequently calculating texture properties.	High accuracy	Tedious operation process	(30)

Compared to glycolytic-type fibers, oxidative-type (Type I and Type IIa) muscle fibers are characterized by their smaller diameters, reduced cross-sectional areas, and higher density, all of which display an inverse relationship with shear force (31). The reverse pattern is seen, however, in glycolytic-type fibers (Type IIx and Type IIb) (32). An elevated proportion of glycolytic Type IIb muscle fibers within the muscle contributes to increased shear force and reduced meat tenderness (33). Therefore, the muscle fiber diameter demonstrates a positive correlation with shear force and a negative correlation with meat tenderness (34). Hence, enhancing meat tenderness and quality can be achieved by increasing the proportion of Type I muscle fibers.

#### 2.2.5 Flavor

During the processing and production of livestock and poultry meat, substances within the tissues undergo a series of transformations and reactions, ultimately shaping the distinctive flavor profile of the meat product. The unique flavor of a meat product is mainly influenced by the phospholipid content within the muscle. Oxidative-type muscle fibers typically contain higher phospholipids levels compared to glycolytic-type muscle fibers, yielding a more robust and aromatic flavor (35). This observation indicates a direct relationship between the proportion of oxidative-type fiber in muscle tissue and the meat flavor. Due to technological advancements, methods for evaluating meat quality are becoming increasingly objective. For example, electronic nose and tongue. Electronic nose is an electronic scanning instrument that simulates human olfaction for odor analysis and judgment. An electronic nose is a scanning device that mimics human olfaction to analyze and assess odors. Notably, it has the characteristics of non-destructive testing, fast analysis, simple operation, and accurate results. It is often used for the classification of varieties, safety inspections, and processing control of meat from livestock and poultry (36). A previous study found that it was a significant difference of quality and flavor among Beijing-you chicken, Luhua chicken and Arbor Acres broiler via electronic nose analysis, and the variety and relative content of aldehydes might contribute to the Beijing-you chickens' richer flavor (37). The electronic tongues simulate human receptor mechanism, detect the sample information by sensor array, can analyze, identify, and judge the tested sample, and process the obtained data using multivariate statistical methods to quickly reflect the overall quality information of the sample (38). Through the use of electronic tongue measurement analysis, 5′-AMP and carnosine were confirmed as the key taste-active compounds contributing to the taste perception of the chicken soup (39). Recent studies have proposed an evaluation index for flavor substances in pork, providing a theoretical support for a more accurate assessment of meat flavor (40).

## 3 Fiber type composition of skeletal muscle and its relationship to meat quality

### 3.1 Skeletal muscle fiber types

Skeletal muscle represents a substantial portion of animal body, comprising approximately 40% of the total mass and closely associated with metabolic functions (41). Muscle fibers can be classified into different types based on various criteria. One criterion is the speed of the contraction, which divides muscle fiber into two categories: slow-twitch (Type I) and fast-twitch (Type II) fibers (42). Currently, the most prevalent classification method relies on the composition of myosin heavy chain (MyHC) isoforms within muscle fiber, including slow oxidative fibers (Type I), fast oxidative fibers (Type IIa), intermediate fibers (Type IIx), and fast glycolytic fibers (Type IIb) (43). Experimental findings have demonstrated that high-frequency electrical stimulation or hyperthyroidism can induce a muscle transformation: Type I → Type IIa → Type IIx → Type IIb, while low-frequency electrical stimulation or hypothyroidism can lead to an opposite transformation: Type IIb → Type IIx → Type IIa → Type I (44). Notably, these transformations are neither obligatory nor universally applicable across different species (45). Moreover, these transformations are triggered by specific conditions and are regulated by multiple signaling pathways, genes, and related cytokines. With the changes in the age, nutrition intake and environment, muscle fibers would undergo phenotypic transformation to adapt to external requirements. Essentially, skeletal muscles alter their fiber composition to impact muscle performance and metabolism (46). The composition of muscle fiber types varies among different breeds. For example, the oxidative fiber content is significantly higher in local Chinese Jinhua pigs compared to imported Landrace pigs (47). However, there is a scarcity of research regarding the composition of muscle fiber types in poultry and its subsequent effects on meat quality.

### 3.2 Relationship between skeletal muscle fiber types and meat quality

Different muscle fiber types directly influence the color and tenderness of both livestock and poultry meat, establishing a strong connection between the quality of fresh meat and the composition of muscle fiber types (48). Muscles with a higher proportion of oxidative Type I and Type IIa muscle fibers display characteristics such as a vibrant red color, lower shear force, smaller fiber size, higher fiber density, enhanced tenderness, higher phospholipid content, and a more pronounced flavor (49). Conversely, muscles with a higher prevalence of Type IIb fibers exhibit a paler and coarse appearance (5). Study has demonstrated a negative correlation between muscle fiber diameter and tenderness, indicating that smaller fiber diameter and greater fiber density contribute to a finer texture in the meat (31). Research has also indicated that Type IIb muscle fibers possess the highest glycolytic capacity, and the rate of pH decline after animal slaughter is positively correlated with both the muscle's glycolytic capacity and the proportion of Type II fibers, particularly Type IIx and Type IIb fibers (50). Additionally, a decrease in phospholipid content within Type IIb muscle fibers can diminish the meat flavor (50). In general, there is a positive correlation between meat quality and the proportion of Type I and Type IIa muscle fibers, while a negative correlation is observed with the proportion of Type IIb muscle fibers (51). Therefore, during the assessment of meat quality, a higher proportion of oxidative slow-twitch muscle fibers (Type I) is generally considered as indicative of superior meat quality. The potential relationship between muscle fiber types and meat quality was listed in Table 4.

**Table 4:** Relationship between skeletal muscle fiber types and meat quality.

**Muscle fiber types**	**Subdivision**	**Characteristic**	**Correlation to meat quality**	**References**
Oxidative fibers	Type I, Type IIa	Muscles exhibit bright red color, lower shear force, smaller fiber size, higher fiber density, enhanced tenderness, increased phospholipid content, and more pronounced flavor characteristics.	Positive	(43, 49)
Glycolytic fibers	Type IIx, Type IIb	Exhibit a paler and coarse appearance; Type IIb muscle fibers have the highest glycolytic ability, causing a rapid decrease in muscle pH value, resulting in contraction of myofibrils within the muscle fibers, leading to a decrease in water content, tenderness, and meat flavor.	Negative	(5, 50)

## 4 Regulators, signaling pathways and molecular mechanisms involved in the regulation of skeletal muscle fiber type in livestock

The development of skeletal muscle in livestock involves a series of intricate processes, including the proliferation and differentiation of myogenic cells, as well as the maturation of myotubes. These processes are meticulously regulated by genes and genes families that associated with muscle development, especially the well-known MRFs family, Pax family, Homeobox Transcription Factor Sine oculis homeobox (Six) family, and the *Mstn* gene. Furthermore, the signaling pathways such as FoxO signaling pathway, the peroxisome proliferator-activated receptor gamma coactivator 1-alpha (PGC-1α) pathway, Wingless/Integrated (Wnt), and sonic hedgehog (Shh) signaling pathways also play significant roles in skeletal muscle development. The following sections will discuss these genes and their underlying molecular mechanisms in their relationship to fast and slow-twitch muscle fiber types.

### 4.1 Genes and transcription factors promoting fast-twitch fiber type

**Figure 1:**
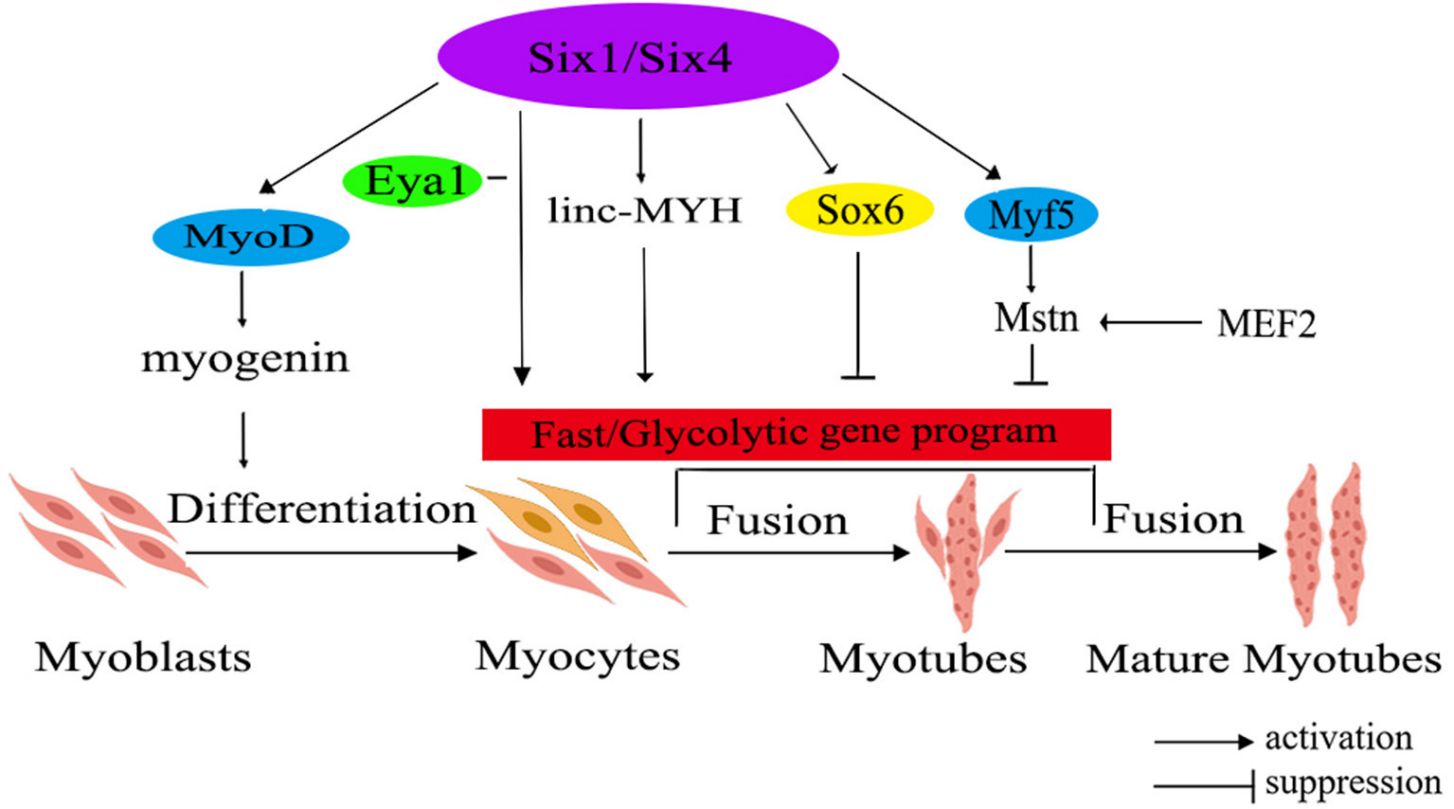
The molecular regulatory network diagram for fast muscle fiber transformation in livestock. The diagram illustrates various gene families using different colors within the ellipse. Additionally, the red rectangle represents a correlation with fast muscle fiber. Six1/Six4, sine oculis homeobox 1/4; MyoD, myogenic determining factor; Eya1, eyes absent 1; linc-MYH, long intergenic non-coding-fast myosin heavy chain; Sox6, SRY-related high-mobility group box 6; Myf5, myogenic factor 5; Mstn, myostatin; MEF2, myocyte-specific enhancer-binding factor 2.

Mstn and Six1 have been identified as crucial regulators contributing to the transformation of fast-twitch muscle fiber. The related regulatory network is shown in Figure 1. Mstn, with its protein primarily secreted within muscle tissues, plays an important role in regulating the growth and differentiation of muscle cells. Notably, research has demonstrated that Mstn has a positive regulatory effect on the proportion of slow-twitch fiber, while exerting a negative impact on the ratio of fast-twitch fiber (52). Recently, it has been unveiled that Six1 transcription factor plays a significant role in skeletal muscle development and the conversion of fiber type. Its regulatory functions extend from the embryonic period through the postnatal stages, indicating the important role that Six1 plays in skeletal muscle (53).

*Mstn* has been found as a negative regulator of skeletal muscle growth. Elevated *Mstn* expression hinders muscle growth, whereas its deficiency or reduced expression leads to muscle hypertrophy or the occurrence of double muscles in animals (54). Research has shown that *in vivo* knockout of the *Mstn* gene in mice leads to an increased fiber diameter and fiber number, leading to the conversion of muscle fibers to the fast-twitch type (55). Study on livestock has indicated that the activation of the porcine *Mstn* promoter is regulated by myocyte-specific enhancer-binding factor 2 (MEF2), whereas the expressions of *Mstn* in cattle and sheep is controlled by myogenic factor 5 (*Myf5*) and myogenic determining factor (*MyoD*) (56).

The Six family is divided into three subfamilies: Six1/2, Six3/6, and Six4/5. Among them, the homologous domain transcription factor Six1 is an upstream regulator of the *MRFs* gene family, including *MyoD*, myogenin (*MyoG*), *Myf5*, and muscle regulatory factor 4 (*MRF4*). Furthermore, Six1 could also regulate the expression of SRY-related high-mobility group box 6 (*Sox6*) and long intergenic non-coding -fast myosin heavy chain (linc-MYH), thereby facilitating the expression of genes associated with fast-twitch muscle (57). It's worth noting that the expressions levels of *Sox6* and linc-MYH are significantly higher in fast-twitch muscles compared to slow-twitch muscles (58). Additionally, the expression of *Six1* exhibits a significant increase in fast-twitch muscles in comparison to slow-twitch muscles (53). Moreover, the co-overexpression of Six1 and its transcription co-activator, eyes absent 1 (Eya1), has been demonstrated to trigger a conversion of slow-twitch oxidative muscle fibers into fast-twitch glycolytic muscle fibers (59). Notably, overexpressing Six1 alone is insufficient to drive this transformation, highlighting a synergistic effect where Six1 and Eya1 collaborate to target downstream genes, leading to the transition from a slow muscle phenotype to a fast muscle phenotype. In instances where Six1 is absent, Six4 and Six5 can selectively compensate for its absence by regulating the transcription of downstream genes associated with fast-twitch muscle (59). Consequently, it can be inferred that Six1, as an upstream regulator of fast-twitch muscle fiber types, collaborates with Eya1 to regulate the transformation of skeletal fiber types. Simultaneously, Six4 and Six5 serve as compensatory mechanisms in the absence of Six1, ensuring the appropriate regulation of genes related to fast-twitch muscle.

### 4.2 Genes, signaling pathway that promoting slow-twitch fiber type

**Figure 2:**
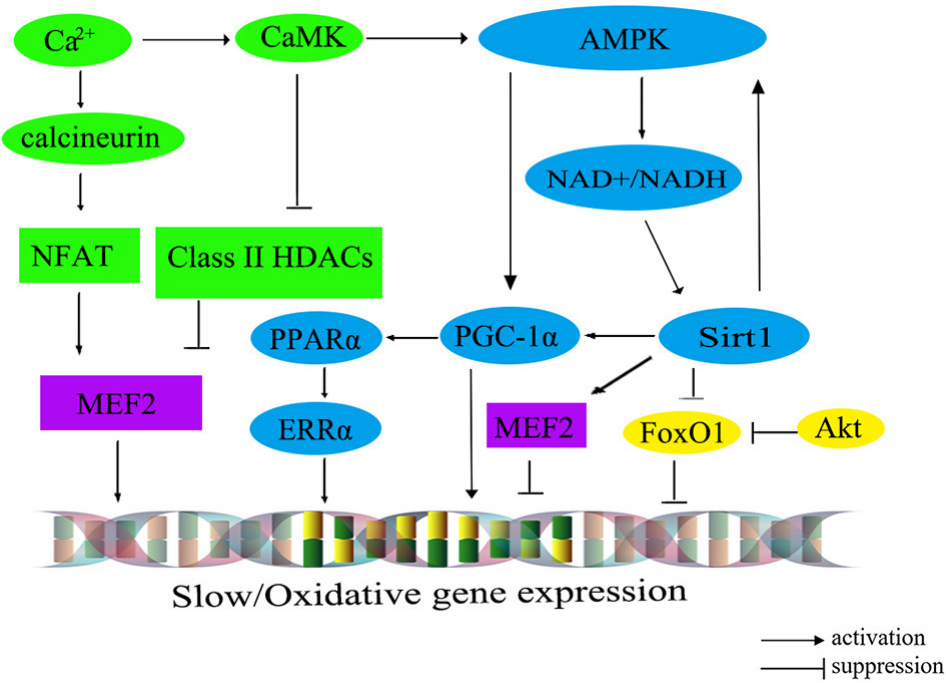
The molecular regulatory network diagram for slow muscle fiber transformation in livestock. The calibration signaling pathway is depicted in green, while the AMPK signaling pathway is represented in blue. The Akt/FoxO1 pathway is visualized in yellow, and transcription factors within the nucleus are portrayed in purple. CaM, calmodulin; CaMK, calmodulin dependent protein kinase; AMPK, AMP-activated protein kinase; Sirt1, silent information regulator 1; PGC-1α, peroxisome proliferator-activated receptor γ co-activator 1α; MEF2, myocyte-specific enhancer-binding factor 2; CaN, calcineurin; NFAT, nuclear factor of activated T cells; FoxO1, fork headbox transcription factor O1; Akt, protein kinase B; PPARα, peroxisome proliferator-activated receptor alphα; ERRα, estrogen-related receptor alphα.

The transition from fast- to slow-twitch fiber types primarily occurs through multiple signaling pathways, such as the Ca^2+^ pathway, the FoxO pathway, and the PGC-1α pathway. These interconnected pathways exert a negative influence on the formation of slow-twitch fibers or form an energy-sensing network by regulating cellular energy metabolism (60). A comprehensive summary of this regulatory network is shown in Figure 2 (61).

The calcium ions (Ca^2+^) signaling pathway, encompassing the Calcineurin (CaN) and calmodulin dependent protein kinase (CaMK) pathways, plays an important role in the regulation of muscle fiber type. Upon binding, CaMK interacts with the calcium-calmodulin (CaM) complex, leading to the activation of CaN. Subsequently, it prompts the dephosphorylation of the transcription factor NFAT (nuclear factor of activated T cells). Serving as both an activator and suppressor of gene expression, NFAT cooperates with the transcription factor MEF2 in the nucleus to regulate the transcriptional activity of oxidative muscle fiber-related genes, thereby promoting the formation of slow-twitch fibers (62). Additionally, an increase in intracellular Ca^2+^ concentration can trigger the activation of CaMK, which in turn further activates *MEF2* through the phosphorylated histone deacetylases (HDACs), thereby regulating the transcription of type I muscle fiber gene (63). Intriguingly, the elevation of intracellular Ca^2+^ levels could also stimulate the AMP-activated protein kinase (AMPK) pathway, which maintains the energy homeostasis by altering the mitochondrial biogenesis, nicotinamide adenine dinucleotide (NAD^+^) levels, and ATP production, shifting it toward the ATP-generating catabolic state. Consequently, the activation of the AMPK signaling pathway facilitates the transformation of skeletal muscle fiber type from fast-twitch muscle to slow-twitch muscle.

Recent findings have illuminated that AMPK can regulate PGC-1α, a signal transmitter of Ca^2+^ second messengers, regulating the formation of slow-twitch muscle fibers (64). Furthermore, AMPK can also enhance the transcription of PGC-1α by increasing intracellular NAD^+^ levels and activating Sirt1 expression (50). In mice with skeletal muscle overexpressing *Sirt1*, there is an upregulation in PGC-1α expression, leading to the conversion of muscle fiber type from fast-twitch to slow-twitch. However, the knockout of the Sirt1 in mice does not significantly alter the composition of different muscle fiber types (65), suggesting the involvement of other regulatory pathways (indicated by the green and blue colors in Figure 2).

PGC-1α plays a critical role in regulating mitochondrial oxidative metabolism. Its primary effects are mediated through downstream effector proteins, namely peroxisome proliferator-activated receptor alph (PPARα) and estrogen-related receptor alph (ERRα). This regulatory impact extends to muscle fiber types, leading to an increase in both the quantity and size of mitochondria in type I fibers, while type IIb fibers possess fewer and smaller mitochondria. Recent research has demonstrated the ability of PGC-1α to promote the transition from glycolytic to oxidative muscle fibers in porcine skeletal muscle (66). The interplay among AMPK, Sirt1, and PGC-1α has been identified to promote the transformation from fast-twitch to slow-twitch muscle fibers. For example, several pathways have been identified as significant contributors to the transformation of skeletal muscle fibers from type II to type I. These contributors include the AMPK/SIRT1/PGC-1α pathway (67), the Sirt1/AMPK pathway (68), and the AMPK/PGC-1α signaling pathway (69). Moreover, the Sirt1/AMPK/PGC-1α signaling pathway also has been demonstrated to facilitate the transformation of type II muscle fibers into type I muscle fibers in post-weaning piglet (70) (indicated by the blue colors in Figure 2).

Furthermore, the protein kinase B (Akt)/FoxO1 pathway has been associated with the regulation of muscle fiber types (71), which has been shown to negatively regulate the proliferation of bovine muscle cells (72). Notably, research on cattle has demonstrated a noteworthy inverse relationship between the expression levels of *FoxO1* and *FoxO4* and muscle fiber area (73). For instance, the expression level of *FoxO4* exhibits a significantly negative correlation with fiber diameter, while *FoxO1* negatively regulates the expression of genes related to type I muscle fiber (73) (indicated by the yellow colors in Figure 2). However, the expression level of *FoxO3* is significantly correlated with muscle fiber density, fiber area, and fiber diameter (73).

## 5 Regulatory mechanisms involved in the poultry skeletal muscle fiber type

Traditional gene function research has illuminated the involvement of *MRFs, Mstn*, and various signaling pathways in the fundamental processes regulating skeletal muscle development. However, with the advancement of novel technologies and research methods, it has become evident that non-coding RNAs, including microRNAs (miRNAs), long non-coding RNAs (lncRNAs), and circular RNAs (circRNAs), also play pivotal roles in skeletal muscle development (74). The following section will categorizes and briefly introduces the relevant genes related to the regulation of skeletal muscle fiber types in poultry.

### 5.1 Genes, miRNAs related to fast-twitch fiber formation in poultry

**Figure 3:**
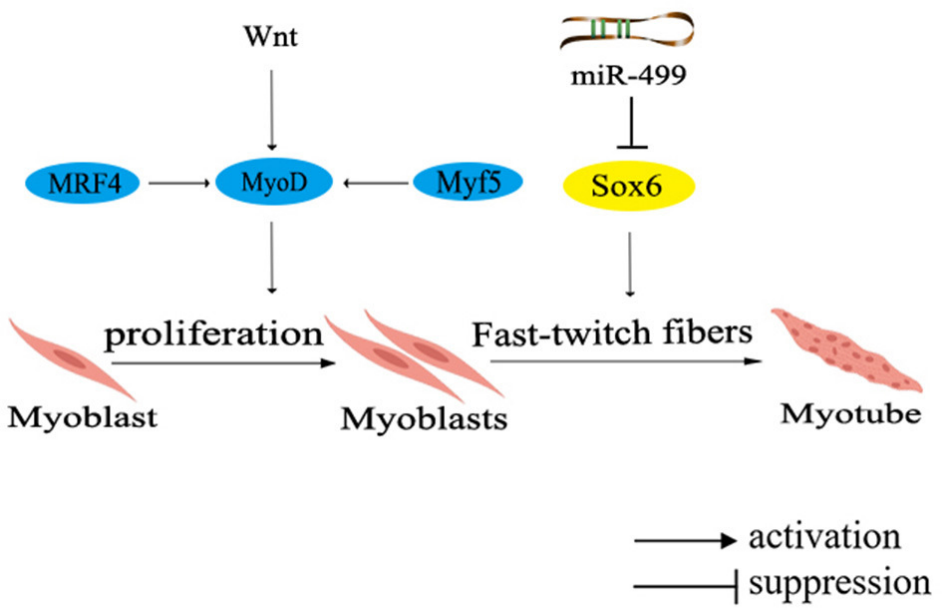
Network diagram of regulating fast muscle fiber types in poultry. Different colors in the ellipse represent different gene families. MRF4, muscle regulatory factor 4; Wnt, Wingless/Integrated pathway.

In the muscle development of poultry, certain genes have been identified to promote the fast-twitch fiber type, including *MRF4, Wnt4, Wnt11*, and *Sox6*. Among them, *MRF4*, a member of the MRFs family, participates in muscle cell determination and differentiation by regulating the expression of muscle-specific proteins during specific stages of muscle differentiation. Collaboration among members of the MRFs family triggers the differentiation and maturation of precursor myoblasts into muscle fibers (75). On a different note, both Wnt4 and Wnt11 belong to the Wnt family of secreted glycoproteins, which can activate the intracellular signaling pathways and regulate target gene transcription by binding to receptors through either paracrine or autocrine mechanisms (76). Another significant contributor, *Sox6*, a member of the SRY-related high-mobility group box (Sox) transcription factor D subfamily, was unveiled to promote muscle differentiation (77). Collectively, these genes orchestrate the regulatory network governing fast-twitch fiber types, as shown in Figure 3.

The expression of *MRF4* is activated during early embryonic development and subsequently initiates the expression of *MyoD*. Collaboratively, MyoD and Myf5 cooperate to regulate muscle cell proliferation and precisely control muscle fiber hypertrophy, diameter, and type. Remarkably, in chicken muscle, the expression of *MRF4* is primarily regulated by Myf5 after hatching, and then participates in the formation of secondary muscle fibers, thereby modulating the regulation of muscle development. Research indicates that Wnt4 and Wnt11 promote the formation of fast-twitch fibers during the early stages of chicken muscle development (78). Further investigation have revealed that Wnt4 upregulates the expression of *Myf5* and *MRF4*, while inhibits *Mstn* expression through the canonical Wnt signaling pathway, enabling differentiated myoblasts to fuse into muscle fibers and promoting muscle fiber hypertrophy. However, a noteworthy discovery has shown that Wnt4 can counteract the transduction of canonical Wnt signaling transduction in C2C12 cells via a non-classical Wnt signaling pathway (76). Although the precise mechanism underlying the regulation of Wnt4 remains controversial, it is indisputable that elevating *Wnt4* expression leads to an increased count of fast-twitch muscle fibers in chicken embryos.

Moreover, muscle-specific miRNAs, located in the introns of *MYH* gene, have been found to participate in the regulation of muscle fiber types. Notably, miR-499 has been unvealed to negatively regulate the formation of fast-twitch muscle, inhibited the formation of fast-twitch muscle fibers through repressing the expression of *Sox6* (Figure 3) (77). It has been found that the knockout of *Sox6* can induce remodeling of muscle fiber types and effectively regulate muscle metabolism (79). Furthermore, deeper exploration has demonstrated that *Sox6* directly inhibits the transcription of genes related to slow-twitch muscle fiber by binding to the conserved cis-regulatory elements (46). Additionally, *Sox6* could also promote the differentiation of myoblasts, the formation of muscle tubes and the fast-twitch muscle fibers (80).

Intriguingly, researches have found that the pectoral muscles of chickens, ducks, and geese all contain fast-twitch muscle fiber composition (31, 81, 82). Duck research has provided genetic evidence supporting the involvement of taspase 1 (*TASP1*) gene, which contributed to increased duck breast muscle fiber diameter (83). Interestingly, it was demonstrated that *MSTN* mRNA expression displayed the highest abundance in the breast muscle than that in other tissues of pigeons, and its expression level exhibited significantly positive correlation with muscle fiber diameter (84).

### 5.2 Non-coding RNAs related to slow-twitch fiber type in chicken

**Figure 4:**
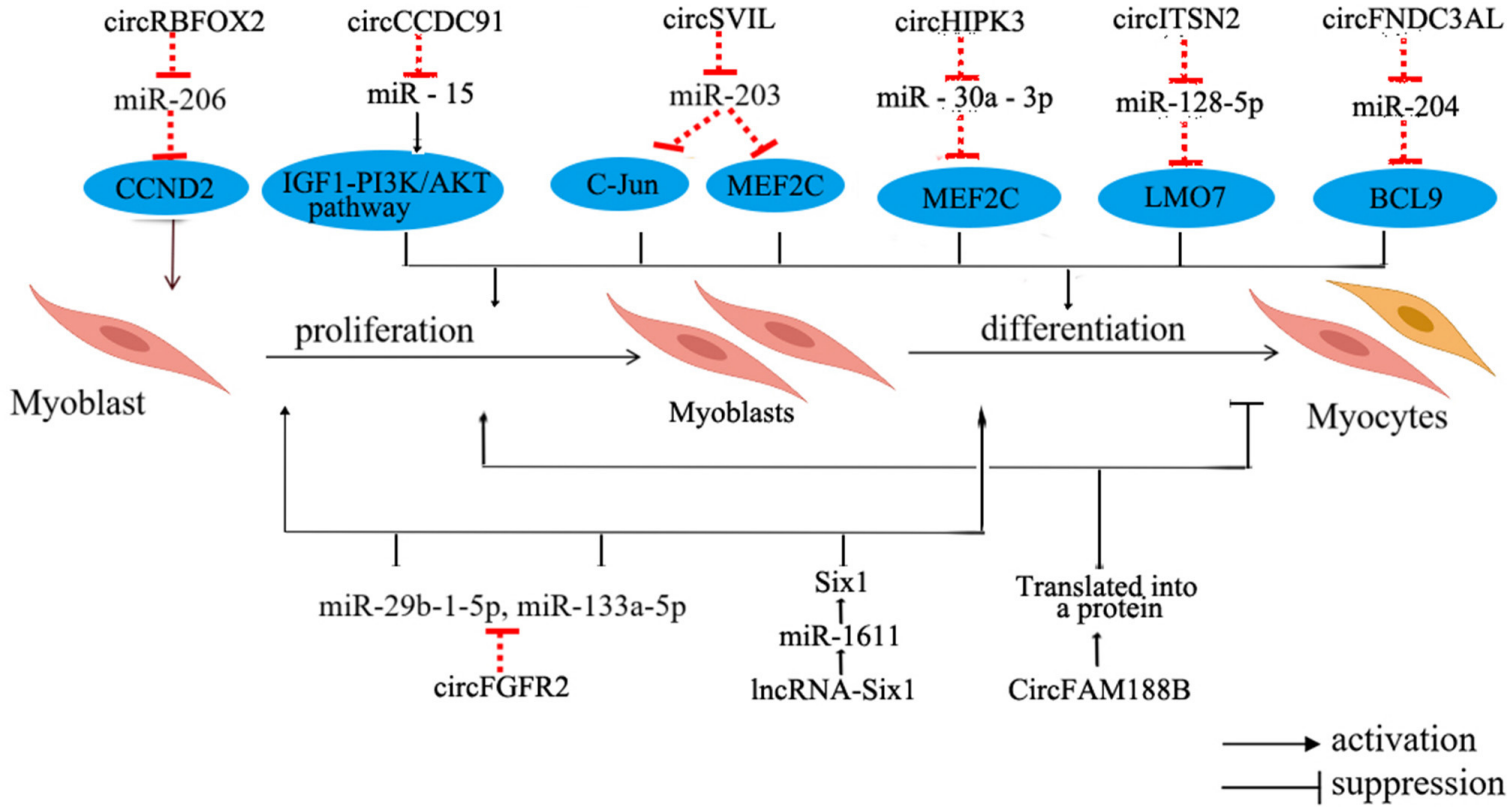
Regulation of non-coding RNA in chicken skeletal myogenesis. The blue oval represents the target gene. LMO7, LIM domain 7; BCL9, B-cell CLL/lymphoma 9.

With the advancement of technology, researchers have discovered numerous non-coding RNAs that play a role in regulating the growth and development of skeletal muscles in chicken (85) (Figure 4).

CircRNAs in skeletal muscle mainly supervise the regulation of miRNA and downstream pathways within skeletal muscle. These circRNAs serve as competitive adsorbents for miRNAs, exerting their influence through various mechanisms. For instance, circSupervillin (circSVIL) in chickens functions as a molecular sponge for miR-203, resulting in the increased expression of *c-Jun* and Myocyte enhancer factor 2C (*MEF2C*), subsequently promoting the proliferation and differentiation of chicken myoblasts (86). Another example is circRNA of RNA binding protein fox-1 homolog 2s (circRBFOX2s), which increases the expression of Cyclin D2 (*CCND2*) through adsorbing miR-206, further promoting cell proliferation (87). Moreover, circHIPK3 can adsorb miR-30a-3p, which binds to the target gene *MEF2C*, thereby promoting the proliferation and differentiation of myoblasts (88). Remarkably, circRNAs exhibit differential expression patterns in oxidative and glycolytic muscles, implying their potential role in regulating the development of distinct skeletal muscles types and participating in the regulation of muscle fiber type transition (89). However, the intricate molecular mechanisms underlying these processes require further investigation.

Concerning the regulatory mechanisms of lncRNAs in the growth and development of chicken skeletal muscle, current research underscores their roles as competing endogenous RNAs (ceRNAs). These lncRNAs function by adsorbing miRNAs and small molecule peptides to regulate the expression of genes related to skeletal muscle development and participate in cell differentiation. For instance, miR-1611 contains potential binding sites for both *lncRNA*-*Six1* and *Six1*. This unique configuration allows *lncRNA-Six1* to competitively bind to miR-1611, subsequently modulating the expression of *Six1*. The overexpression of this gene inhibits myoblast proliferation and differentiation (90). Furthermore, this study also highlights that miR-1611 exhibits heightened expression levels in slow-twitch fibers, contributing to the transformation of fast-twitch fibers into slow-twitch fibers. Additionally, in chickens, miR-499 suppresses the expression of *Sox6*, thereby facilitating the formation of slow-twitch fibers (91). These findings collectively emphasize the significant contribution of non-coding RNAs to the intricate process of skeletal muscle development and the regulation of muscle fiber types.

## 6 Conclusion

Previous researches mentioned above have demonstrated that a higher proportion of oxidative slow-twitch muscle fibers (Type I) in the muscles of livestock and poultry is associated with superior meat quality. Intentionally increasing the presence of slow-twitch muscle fibers in livestock and poultry can effectively improve economic benefits in practical production and farming. For example, Six1, a transcription factor involved in muscle progenitor cell development, skeletal muscle growth, and muscle fiber types transformation. Six1 plays a pivotal role in skeletal muscle development and serves as a focal gene for investigating the regulation of livestock growth. Although there have been advancements in the understanding of molecular regulatory mechanisms governing skeletal muscle development, some intricate regulatory processes still lack complete understanding. As our comprehension of the regulatory mechanisms that control skeletal muscle fiber types continues to expand, the potential to improve the meat quality of livestock and poultry through molecular breeding approaches becomes more achievable.

## Author contributions

MM: Writing—original draft. ZZ: Writing—original draft, review & editing. XW: Investigation, Writing—review & editing. WS: Writing—review & editing. LZ: Supervision, Writing—review & editing. SL: Supervision, Writing—review & editing.
